# *EGFR*-Mutated Squamous Cell Lung Cancer and Its Association With Outcomes

**DOI:** 10.3389/fonc.2021.680804

**Published:** 2021-06-14

**Authors:** Rui Jin, Ling Peng, Jiawei Shou, Jin Wang, Yin Jin, Fei Liang, Jing Zhao, Mengmeng Wu, Qin Li, Bin Zhang, Xiaoying Wu, Fen Lan, Lixia Xia, Junrong Yan, Yang Shao, Justin Stebbing, Huahao Shen, Wen Li, Yang Xia

**Affiliations:** ^1^ Key Laboratory of Respiratory Disease of Zhejiang Province, Department of Respiratory and Critical Care Medicine, Second Affiliated Hospital of Zhejiang University School of Medicine, Hangzhou, China; ^2^ Department of Respiratory Disease, Zhejiang Provincial People’s Hospital, Hangzhou, China; ^3^ Department of Radiotherapy, First Affiliated Hospital, School of Medicine, Zhejiang University, Hangzhou, China; ^4^ Department of Medical Oncology, Sir Run Run Shaw Hospital, College of Medicine, Zhejiang University, Hangzhou, China; ^5^ Institute of Cancer and Basic Medicine (ICBM), Chinese Academy of Sciences, Department of Medical Oncology, Cancer Hospital of the University of Chinese Academy of Sciences, Hangzhou, China; ^6^ Institute of Cancer and Basic Medicine (ICBM), Chinese Academy of Sciences, Department of Radiation Oncology, Cancer Hospital of the University of Chinese Academy of Sciences, Hangzhou, China; ^7^ Department of Biostatistics, Zhongshan Hospital, Fudan University, Shanghai, China; ^8^ Department of Medical Oncology, Second Affiliated Hospital of Zhejiang University School of Medicine, Hangzhou, China; ^9^ Translational Medicine Research Institute, Geneseeq Technology Inc., Toronto, ON, Canada; ^10^ Division of Cancer, Department of Surgery and Cancer, Imperial College London, London, United Kingdom

**Keywords:** lung squamous cell carcinoma, epidermal growth factor receptor, tyrosine kinase inhibitor, genomic profile, progression-free survival

## Abstract

**Background:**

The therapeutic efficacy of epidermal growth factor receptor tyrosine kinase inhibitors (EGFR-TKIs) in advanced *EGFR*-mutant lung squamous cell carcinoma (SCC) patients remains uncertain. Furthermore, the factors underlying the responsiveness have not been fully investigated. We therefore investigated the link between genomic profiles and EGFR-TKI efficacy.

**Material and Methods:**

We consecutively enrolled stage IV, *EGFR*-mutant, and EGFR-TKI–treated patients with SCC. Patients with *EGFR* wild-type lung SCC and *EGFR*-mutant lung adenocarcinoma were consecutively enrolled as controls, and next-generation sequencing (NGS) was performed.

**Results:**

In total, 28 *EGFR*-mutant lung SCC, 41 *EGFR*-mutant lung adenocarcinoma, and 40 *EGFR* wild-type lung SCC patients were included. Among the patients with *EGFR* mutations, shorter progression-free survival (PFS) was observed in SCC compared to adenocarcinoma (4.6 *vs.* 11.0 months, P<0.001). Comparison of the genomic profiles revealed that *EGFR*-mutant SCC patients had similar mutation characteristics to *EGFR*-mutant adenocarcinoma patients, but differed from those with *EGFR* wild-type SCC. Further exploration of *EGFR*-mutant SCC revealed that mutations in *CREBBP* (P = 0.005), *ZNF217* (P = 0.016), and the Wnt (P = 0.027) pathway were negatively associated with PFS. Mutations in *GRM8* (P = 0.025) were associated with improved PFS.

**Conclusions:**

*EGFR*-mutant lung SCC has a worse prognosis than *EGFR*-mutant adenocarcinoma. Mutations in other genes, such as *CREBBP*, *ZNF217*, *GRM8*, or Wnt that had implications on PFS raise the possibility of understanding mechanisms of resistance to EGFR-TKI in lung SCC, which will aid identification of potential beneficial subgroups of patients with *EGFR*-mutant SCCs receiving EGFR-TKIs.

## Introduction

Epidermal growth factor receptor (EGFR) represents as the most frequently mutated driver gene in lung cancer. In comparison with lung adenocarcinoma, *EGFR* mutations are relatively rare lung squamous cell carcinoma (SCC), with a reported prevalence of 3% to 18% (1–10). In light of this, the therapeutic value of targeted therapy, EGFR tyrosine kinase inhibitors (EGFR-TKIs), in advanced lung SCC patients lacks in-depth multiple dimensional exploration with large cohorts. Pilot studies have reported moderate effectiveness of EGFR-TKIs in *EGFR*-mutant lung SCC, with an objective response rate (ORR) ranging from 25% to 49% (11–17). However, shortened progression-free survival (PFS) was shown, ranging from 1 to 5 months, in *EGFR*-mutant SCC patients (11–15, 17–21). Still, evidences derived from large-scale prospective cohorts are lacking.

Furthermore, the mechanism underlying the limited efficacy of EGFR-TKIs in *EGFR*-mutant lung SCC is poorly understood. Data from the Cancer Genome Atlas (TCGA) and Chinese cohorts have revealed that lung SCC exhibited a different genomic profile from that of lung adenocarcinoma (4, 22–24), providing insights into the study involving genome-based efficacy analysis. Regrettably, there are currently no studies exploring the genomic profile of *EGFR*-mutant lung SCC or analyzing the association between the genomic features and therapeutic efficacy.

In this study, we retrospectively recruited advanced lung SCC patients with *EGFR* mutations, and enrolled patients with *EGFR*-mutant adenocarcinoma and *EGFR* wild-type SCC. We aimed to characterize the genomic patterns of *EGFR*-mutant SCC, and analyze EGFR-TKI efficacy in *EGFR*-mutant SCC according to the genomic profiles of the patients.

## Materials and Methods

### Patients and Study Procedure

We enrolled 28 *EGFR*-mutant lung SCC, 41 *EGFR*-mutant lung adenocarcinoma, and 40 *EGFR* wild-type lung SCC patients from the Second Affiliated Hospital of Zhejiang University School of Medicine, the First Affiliated Hospital of Zhejiang University School of Medicine, and Sir Run Run Shaw Hospital of Zhejiang University School of Medicine from June 2015 to June 2019. Primary eligibility criteria included histologically confirmed SCC, stage IV non-small cell lung cancer (NSCLC) with *EGFR* mutations, and treatment with EGFR-TKIs. Other eligibility criteria included an Eastern Cooperative Oncology Group (ECOG) performance status of 0 to 2, at least one measurable lesion, and a life expectancy of 3 months or longer. *EGFR*-mutant adenocarcinoma patients and *EGFR*-wild-type SCC patients were consecutively enrolled at the same time.

All patients were diagnosed *via* percutaneous and transbronchial lung biopsy. Pathological diagnosis was confirmed by light microscopy and immunohistochemistry (IHC), and verified by staining for P40 (+) together with thyroid transcription factor (TTF)-1 (−) and napsin A (−). Formalin-fixed paraffin-embedded (FFPE) blocks or 10 to 15 PPFE slices with a thickness of 6 to 10 µm were obtained and the NGS testing was carried out in Nanjing Geneseeq Technology Inc (Nanjing, China) testing laboratory. Samples with tumor cell content above 20% were considered qualified.

The studies involving human patients were reviewed and approved by institutional review board of Second Affiliated Hospital of Zhejiang University School of Medicine.

### Clinical Assessments and End Points

Tumor response was assessed using Response Evaluation Criteria in Solid Tumors (RECIST) version 1.1. The treatment response was evaluated 1 month after the initiation of EGFR-TKI therapy and every 2 to 3 months thereafter, based on the patients’ computed tomography (CT) and magnetic resonance imaging (MRI) data.

The primary outcome was PFS, defined as the time from the start of treatment to disease progression, as confirmed by radiologic diagnosis or death from any cause. Patients who did not relapse or not die were censored at the last follow-up. Exploratory analyses included comparing the genomic profiles between *EGFR*-mutant adenocarcinoma and *EGFR*-mutant SCC patients, and *EGFR*-mutant SCC and *EGFR* wild-type SCC patients. The response to TKIs of *EGFR*-mutant SCC patients according to genomic profile was also analyzed.

### DNA Extraction, Sequencing, and Bioinformatics Analysis

DNA extraction, sequencing library preparation, and targeted capture were carried out following previously described methods, with some modifications (25) (see **Supplementary Methods**). Genomic DNA was extracted using the QIAamp DNA FFPE Tissue Kit (Qiagen), and libraries were prepared by KAPA Hyper Prep kit (KAPA Biosystems). Customized xGen lockdown probes panel (Integrated DNA Technologies) were used to selectively enrich for 425 predefined cancer-related genes (Geneseeq Prime panel) (see **Table S1**). Target-enriched libraries were sequenced on the HiSeq4000 platform (Illumina). Gene fusions were identified by FACTERA, copy number variations (CNVs) were analyzed with ADTEx, and allele-specific CNVs were analyzed by FACETS. Chromosome instability score (CIS) was defined as the proportion of the genome with aberrant (purity-adjusted segment-level copy number >=3 or <=1) segmented copy number. Tumor mutation burden (TMB) was defined as the number of somatic, coding, base substitution, and indel mutations per megabase of genome examined. Briefly, all base substitutions, including non-synonymous and synonymous alterations, and indels in the coding region of targeted genes were considered with the exception of known hotspot mutations in oncogenic driver genes and truncations in tumor suppressors. Synonymous mutations were counted in order to reduce sampling noise, and known driver mutations were excluded as they are over-represented in the panel. The summary of genomic aberrations among three cohorts is presented in **Table S2**.

### Statistical Analysis

Quantitative data are presented as the median (range) with percentages. Comparisons of proportions between groups were performed using Fisher’s exact test. For the survival analysis, Kaplan-Meier curves were generated, and p-values were determined with the log-rank test. Hazard ratios (HRs) were calculated by Cox proportional hazards model. A two-sided p-value of <0.05 was considered significant for all tests unless indicated otherwise. Univariable Cox regression was used to study the association between the different variables and PFS, and the results are presented as HRs with 95% confidence intervals (CIs). All analyses were performed with R 3.6.0 (R Development Core Team).

## Results

### Patient Characteristics

**Figure 1:**
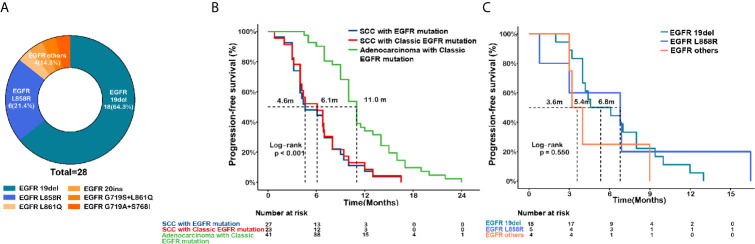
*EGFR* mutational subtypes and survival analysis in *EGFR*-mutated lung squamous cell carcinoma (SCC) and adenocarcinoma. **(A)** Mutation subtypes of *EGFR*-mutant lung SCC. **(B)** Kaplan-Meier curves of progression-free survival (PFS) comparing the SCC patients harboring *EGFR* mutation (n=27, blue) or classic *EGFR* mutation (19del or L858R, n=23, red), and in adenocarcinoma patients harboring classic *EGFR* mutation (19del or L858R, n=41, green). **(C)** Kaplan-Meier curves of PFS in SCC patients harboring 19del (n=18, green), L858R (n=5, blue), or other uncommon mutations (n=4, orange).

Baseline characteristics of the *EGFR*-mutant lung SCC (*n* = 28), *EGFR*-mutant lung adenocarcinoma (*n* = 41), and *EGFR* wild-type lung SCC (*n* = 40) groups were listed in **Table 1**. Among lung SCC patients harboring the *EGFR* mutation, we identified 18 *19Del* (64.3%), 6 *L858R* (21.4%), 1 *L861Q* (3.6%), 1 *20ins* (3.6%), 1 *G719S*+*L861Q* (3.6%), and 1 *G719S*+*S768I* (3.6%) (**Figure 1A**). Among the *EGFR*-mutant adenocarcinoma patients, 19 (64.3%) harbored *19Del*, and 22 (53.7%) harbored *L858R* changes. In patients with *EGFR*-mutant SCC, 27 (96.4%) were treated with first- or second-generation EGFR-TKIs, and only one (3.6%) with osimertinib. Similarly, the vast majority of adenocarcinoma patients (39, 95.1%) received first- or second-generation EGFR-TKIs, only two (4.9%) received osimertinib.

**Table 1:** Baseline clinical characteristics of the study population.

Characteristics	*EGFR*-mutant SCC(N=28)	*EGFR*-mutant adenocarcinoma(N=41)	*EGFR* wild-type SCC (N=40)
**Age**	65 (46–83)	64 (40–80)	67 (50–87)
** ≥65**	13 (46.4%)	21 (51.2%)	35 (87.5%)
** <65**	15 (53.6%)	20 (48.8%)	15 (37.5%)
**Gender**			
** Male**	16 (57.1%)	16 (39.0%)	19 (47.5%)
** Female**	12 (42.9%)	25 (61.0%)	21 (52.5%)
**Smoking status**			
** Ever-smokers**	10 (35.7%)	19 (46.3%)	28 (70.0%)
** Never-smokers**	18 (64.3%)	22 (53.7%)	12 (30.0%)
***EGFR* Mutant**			
** 19Del**	18 (64.3%)	19 (46.3%)	–
** L858R**	6 (21.4%)	22 (53.7%)	–
** Other mutation**	4 (14.3%)	0 (0%)	–
**EGFR-TKI**			
** Icotinib**	14 (51.9%)	18 (43.9%)	–
** Gefitinib**	6 (22.2%)	18 (43.9%)	–
** Erlotinib**	3 (11.1%)	3 (7.3%)	–
** Afatinib**	3 (11.1%)	0 (0%)	–
** Osimertinib**	1 (3.7%)	2 (4.9%)	–

EGFR, epidermal growth factor receptor; TKI, tyrosine kinase inhibitor.

### Efficacy of TKIs for *EGFR*-Mutant SCC and Adenocarcinoma

Among *EGFR*-mutant lung cancer patients receiving EGFR-TKIs, shorter PFS was observed in SCC compared to adenocarcinoma, (median PFS (mPFS): 4.6 *vs.* 11 months, P<0.001, **Figure 1B**). Non-significant differences in PFS were found among the various *EGFR* mutation types in SCC (mPFS: 5.4 months for *19Del vs.* 6.8 months for *L858R vs.* 3.6 months for other mutations, P = 0.550, **Figures 1C**). Also, PFS was not affected by gender (P = 0.56) or smoking status (P = 0.14, **Figure S1A, B**). These findings support the idea that, compared to *EGFR*-mutant adenocarcinoma, SCC with the *EGFR* mutation is less responsive to EGFR-TKIs.

### Construction of Genomic Profiles and Molecular Features Correlated With Blunted Efficacy of *EGFR*-TKI in SCC

#### Comparison of Genomic Profiles Among *EGFR*-Mutant Adenocarcinoma, *EGFR*-Mutant SCC, and *EGFR* Wild-Type SCC

**Figure 2:**
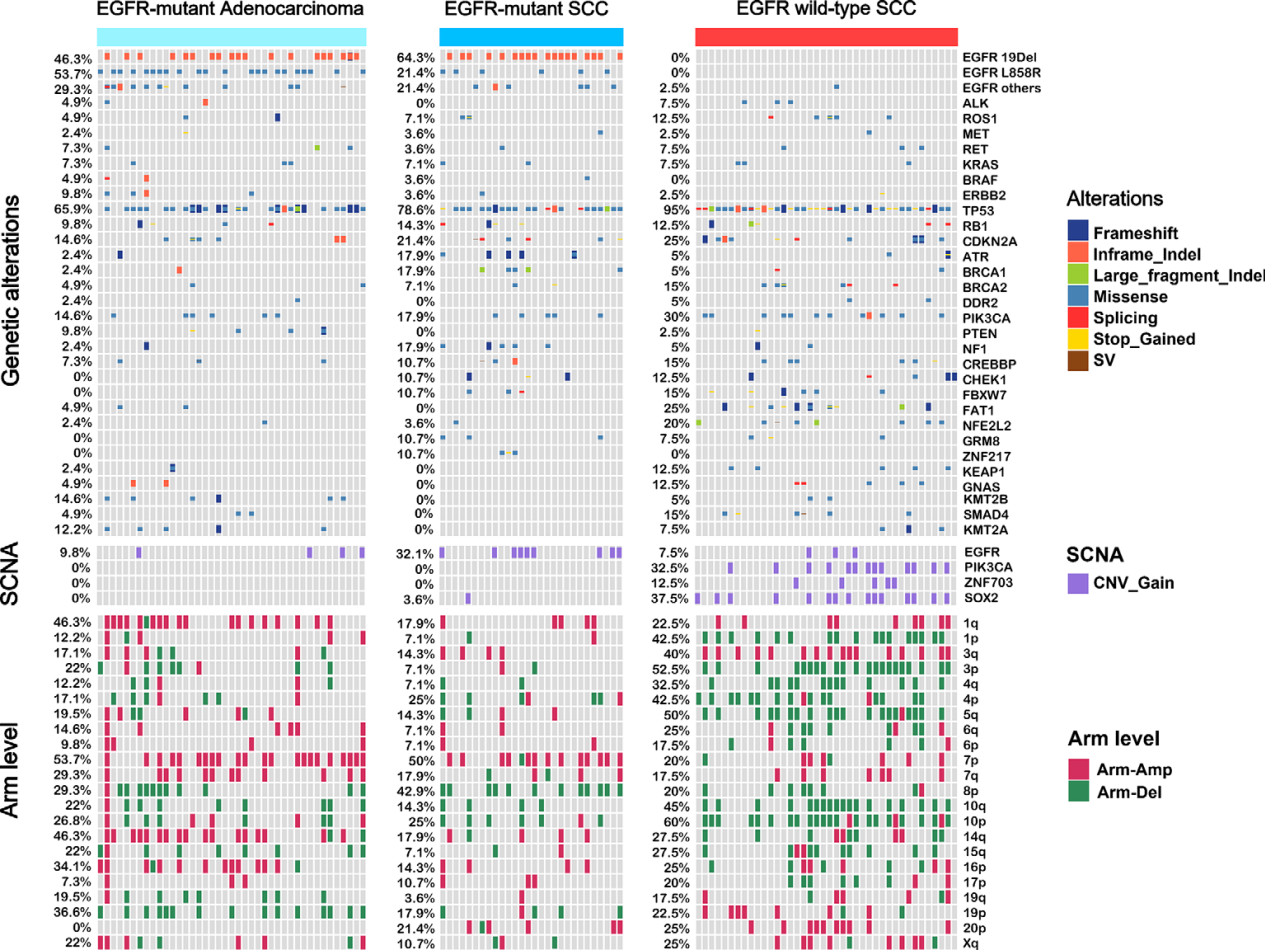
The landscape of genomic profiles in *EGFR*-mutant adenocarcinoma, *EGFR*-mutant SCC, and *EGFR* wild-type SCC. 32 top-ranking genetic alterations (top panel), 4 somatic copy number alterations (SCNAs, middle panel), and 22 arm level alteration (bottom panel) in three subgroups (*EGFR*-mutant adenocarcinoma, *n* = 28; *EGFR*-mutant SCC, *n* = 41; *EGFR* wild-type SCC, *n* = 40) were represented. Each column represented a sample.

We compared genomic profiles between adenocarcinoma and SCC. **Figure 2** shows the genomic changes detected in *EGFR*-mutant adenocarcinoma, *EGFR*-mutant SCC, and *EGFR* wild-type SCC, including genetic alterations, somatic copy-number alterations, and arm-level alterations.

Compared to *EGFR*-mutant adenocarcinoma, *EGFR*-mutant SCC exhibited a higher mutation *ATR* frequency [odds ratio (OR): 8.44, 95% CI: 0.87–420.30, P=0.037], *BRCA1* (OR: 8.44, 95% CI: 0.87–420.30, P=0.037), and *NF1* (OR: 8.44, 95% CI: 0.87–420.30, P=0.037), and more CNVs of *EGFR* (OR: 4.28, 95% CI: 1.03–21.60, P=0.028) (**Table S3**). We also assessed responsiveness to EGFR-TKI according to the mutation profile. Either in *EGFR*-mutant SCC cohort or *EGFR*-mutant adenocarcinoma cohort, no difference was observed in PFS between mutant group and the wild-type group (**Table S4**, **Figures S1C–E**). Notably, *EGFR*-mutant SCC and adenocarcinoma had a similar mutation frequency for TP53 (78.6% *vs*. 65.9%), where this mutation is known to reduce EGFR-TKI efficacy. However, compared to *EGFR* wild-type SCC, *EGFR*-mutant SCC showed a lower mutation frequency for *FAT1* (OR: 0, 95% CI: 0–0.54, P = 0.004) and *SMAD4* (OR: 0, 95% CI: 0–1.14, P=0.039), a higher CNV of *EGFR* (OR: 5.68, 95% CI: 1.23–36.46, P=0.020), and lower CNVs of *SOX2* (OR: 0.06, 95CI: 0.001–0.47, P=0.001) and *PIK3CA* (OR: 0, 95% CI: 0–0.36, P<0.001) (**Table S3**).

**Figure 3:**
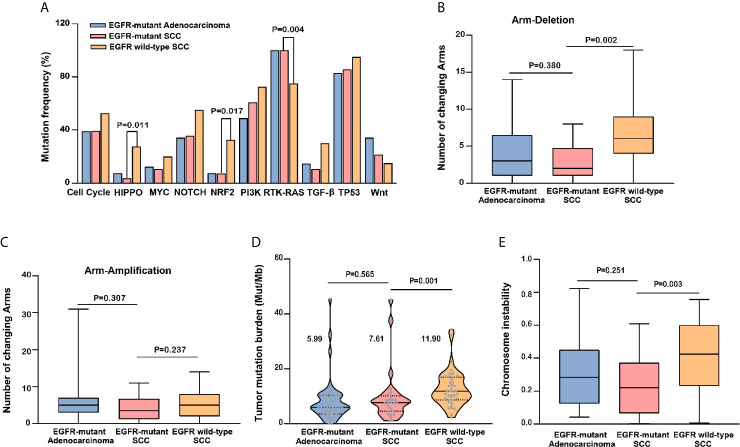
Comparison of genomic profiles among *EGFR*-mutant adenocarcinoma, *EGFR*-mutant squamous cell carcinoma (SCC), and *EGFR* wild-type SCC. Comparison of 10 signaling pathway mutation frequency **(A)**, change number of arm deletion **(B)** and arm amplification **(C)**, tumor mutation burden (TMB) **(D)**, and chromosome instability (CIS) **(E)** among three subsets were represented.

We also assessed signaling pathway involvement. In line with the above results, signaling pathway mutation frequency was identical between *EGFR*-mutant adenocarcinoma and SCC (**Figure 3A** and **Table S5**). In contrast, the HIPPO pathway (OR: 0.10, 95% CI: 0.002–0.77, P=0.011), NRF2 pathway (OR: 0.16, 95% CI: 0.02–0.83, P=0.017), and RTK-RAS pathway (OR: infinity, 95% CI: 1.85–infinity, P=0.004) were significantly different between SCC with versus without the *EGFR* mutation.

Regarding mutation characteristics at the arm-level, the total number of arm deletions (P=0.380, **Figure 3B**) and arm amplifications (P=0.307, **Figure 3C**) was identical between *EGFR*-mutant adenocarcinoma and SCC. In contrast, a lower number of arm deletions (P=0.002, **Figure 3B**) and similar number of arm amplifications (P=0.237, **Figure 3C** **)** were detected in *EGFR*-mutant SCC compared to *EGFR* wild-type SCC.

Finally, we evaluated the TMB and CIS. TMB was comparable between *EGFR*-mutant SCC and adenocarcinoma patients (7.61 *vs*. 5.99 mutations/Mb, P=0.565), the median TMB (mTMB) was 7.61 (95% CI: 5.84–13.40) and 5.99 (95% CI: 5.77–11.13) mutations/Mb, respectively. However, it is interesting that the mTMB was significantly lower in *EGFR*-mutant SCC than EGFR wild-type SCC (7.61 *vs*. 11.9 mutations/Mb, P=0.001), the mTMB was 7.61 (95% CI: 5.84–13.40) and 11.90 (95% CI: 10.72–14.96) mutations/Mb, respectively (**Figure 3D** **).** The CIS was similar between *EGFR*-mutant SCC and adenocarcinoma (P = 0.251). In contrast, the CIS of *EGFR*-mutant SCC was significantly lower than that of *EGFR* wild-type SCC (P = 0.003; **Figure 3E** **)**.

Overall, *EGFR*-mutant SCC showed similar gene characteristics to *EGFR*-mutant adenocarcinoma, but different ones to *EGFR* wild-type SCC.

#### EGFR-TKI Efficacy in *EGFR*-Mutant SCC Patients According to Genetic Profile

To identify the factors influencing EGFR-TKI efficacy in *EGFR*-mutant SCC, PFS was analyzed according to genomic profile within the *EGFR*-mutant SCC cohort.

**Figure 4:**
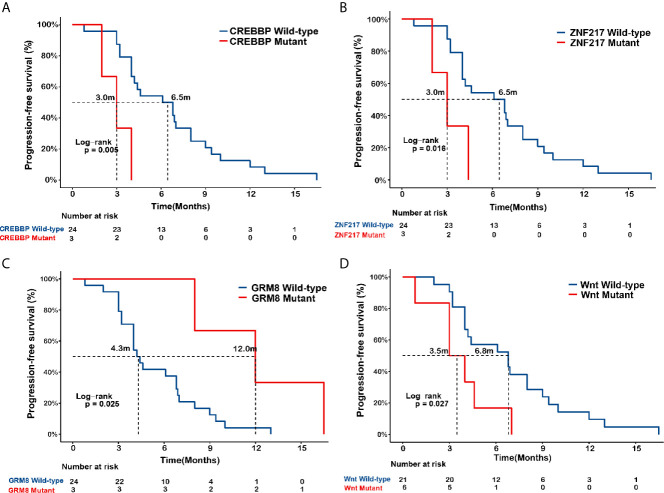
Associations of *CREBBP*, *ZNF217*, *GRM8*, and Wnt pathway mutation with EGFR-TKI outcome in *EGFR*-mutant squamous cell carcinoma (SCC). Kaplan-Meier curves of progression-free survival (PFS) in *EGFR*-mutated lung squamous cell carcinoma (SCC) in terms of mutational status of *CREBBP* **(A)**, *ZNF217* **(B)**, *GRM8* **(C)**, and Wnt pathway **(D)**.

As shown in **Figure 4**, mPFS was poorer in the *CREBBP*-mutant group than the wild-type (3.0 *vs*. 6.5 months, P=0.005) and in the *ZNF217*-mutant SCC group than the wild-type (3.0 *vs*. 6.5 months, P=0.016). Conversely, mPFS was significantly improved in SCC patients with the *GRM8* mutation than the wild-type (12.0 *vs*. 4.3 months, P=0.025). In addition, SCC patients with Wnt pathway mutations exhibited a shorter mPFS than the wild-type (3.5 *vs*. 6.8 months, P=0.027).

**Figure 5:**
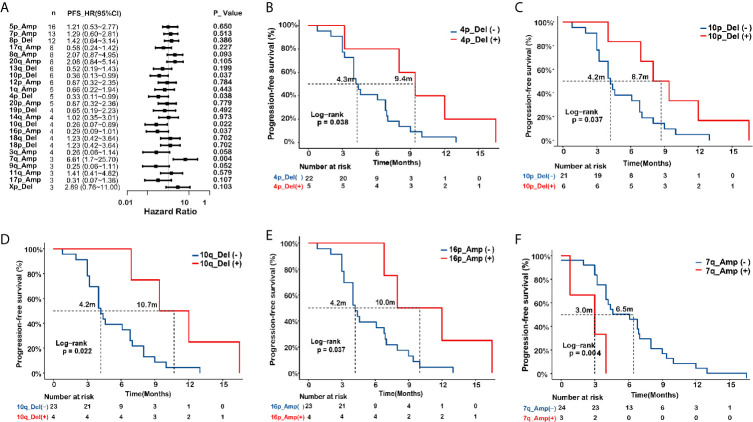
Associations of arm level changes with EGFR-TKI outcome in *EGFR*-mutant squamous cell carcinoma (SCC). **(A)** Forest plot presenting hazard ratios (HRs) of progression-free survival (PFS) comparing subgroups with and without specific arm-level changes in *EGFR*-mutated lung squamous cell carcinoma (SCC). **(B–F)** Kaplan-Meier curves of progression-free survival (PFS) in *EGFR*-mutated SCC in terms of arm-level changes on 4p_Del **(B)**, 10p_Del **(C)**, 10q_Del **(D)**, and 16p_Amp **(E)**, 7q_Amp **(F)**. Del, deletion; Amp, amplification.

Next, we evaluated associations of arm-level changes with the responsiveness to EGFR-TKIs in the *EGFR*-mutant SCC patients. A forest plot is presented in **Figure 5A**. The mPFS was longer in SCC patients with chromosome arm changes on 4p_Del (9.4 *vs*. 4.3 months, P=0.038), 10p_Del (8.7 *vs*. 4.2 months, P=0.037), 10q_Del (10.7 *vs*. 4.2 months, P=0.022), and 16p_Amp (10 *vs*. 4.2 months, P=0.037) (**Figures 5B–E**), whereas mPFS was shorter in the SCC patients with 7q_Amp (3.0 *vs* 6.5 months, P=0.004) (**Figure 5F**).

We also analyzed blunted reactivity to EGFR-TKI in SCC patients according to TMB and CIS. The mPFS did not differ significantly between patients with high and low TMB (8.2 *vs*. 4.2 months, P=0.095), or between patients with CIS ≥ 30% and < 30% (6.1 *vs*. 4.1 months, P=0.351, **Figures S1F–G**). Since smokers were considered to bear higher TMB, this result was in line with the observation in different smoking status subset.

In summary, SCC patients harboring both the *EGFR* and *CREBBP* mutation, and those with the *ZNF217* mutation, Wnt pathway mutation, and 7q_Amp were likely to have a shorter PFS, whereas those with the *GRM8* mutation, or 4p_Del, 10p_Del, 10q_ Del, or 16p_Amp chromosome arm changes, tended to have prolonged PFS.

## Discussion

To our knowledge, this is the first study to explore the therapeutic efficacy of EGFR-TKI in *EGFR*-mutant SCC patients with mutation profiles. In SCC patients harboring the *EGFR* mutation, identifying the factors that influence EGFR-TKI responsiveness, and the subgroups most likely to benefit from these agents, is of great importance. Our study provides evidence of a shorter PFS in *EGFR*-mutant SCC compared to *EGFR*-mutant adenocarcinoma patients. With discrepancy in TKI outcomes, *EGFR*-mutant SCC and adenocarcinoma, however, presented similar genomic patterns. We further observed that specific genomic features, such as *CREBBP*, *ZNF217*, or Wnt pathway mutation, may predict a shortened PFS in SCC patients.

Previous studies have shown that SCC patients with *EGFR* mutations conferred responsiveness to EGFR-TKIs, with a non-inferior ORR (25–49%) to that of lung adenocarcinoma patients (11–16). However, the survival benefit of EGFR-TKIs was not as pronounced in lung SCC patients. Our study confirmed an inferior mPFS (4.6 months) in the *EGFR*-mutant SCC cohort, in line with previous reports of a 1–5 month PFS (20, 26, 27). Previously, patients with lung non-adenocarcinoma, such as large cell lung carcinomas (LCLCs), harboring *EGFR* mutations and treated with EGFR-TKIs, also had a reduced mPFS (4.4 months) (20). In contrast, lung adenosquamous carcinoma patients showed a PFS of 8–14 months (20, 28).

The Cancer Genome Atlas (TCGA) study and data from Chinese lung cancer cohorts have confirmed differences in genomic profiles between lung SCC and adenocarcinoma patients (4, 22–24). In lung SCC, *TP53, NFE2L2, CDKN2A, KEAP1*, and *PTEN* were the most commonly mutated genes. Conversely, in adenocarcinoma, mutations were typically observed in the *TP53, KRAS, EGFR, STK11*, and *RB1* genes. The higher mutation frequency of *TP53* and lower mutation frequency of *EGFR* and *KRAS* were involved in lung SCC. Compared to the TCGA data, the Nanjing Lung Cancer Cohort (NJLCC) (4) and CHOICE cohort (24) of Chinese patients revealed higher rates of *EGFR* and *RB1* mutations, and lower rates of *KRAS, BRAF*, and *STK11* mutations, in adenocarcinoma patients. In SCC patients, there was a higher mutation frequency of *TP53, RB1*, and *NFE2L2*, and a lower mutation frequency of *PIK3CA* and *CDKN2A*. In *EGFR* wild-type SCC patients, *TP53, NFE2L2, CDKN2A, KEAP1, PTEN*, and *RB1* were the most commonly mutated genes, consistent with previously reported genomic profiles.

We observed a higher mutation frequency of *NF1*, *ATR*, and *BRCA1* in *EGFR*-mutant SCC compared to *EGFR*-mutant adenocarcinoma. *NF1* is a tumor suppressor gene that negatively regulates RAS signaling (29). *NF1*‐mutant lung adenocarcinoma patients had inferior disease‐free survival (DFS), and overall survival (OS) compared to those with the *EGFR*-mutation (30), and downregulation of *NF1* expression caused by truncating mutations was reported to confer resistance to EGFR-TKI in lung adenocarcinoma patients (31). Notably, in our study, the *NF1* mutation was negatively, though not significantly, associated with PFS in lung SCC patients, which was possibly due to the relatively small size of the cohort. The ATR kinase encoded by *ATR* is implicated in the DNA damage response (DDR) (32), and is involved in lung cancer development (33). The ATR/CHK1 (the downstream effector kinases of ATR) axis was identified as a potential drug target for small-cell lung cancer (SCLC) patients (34), but no similar study in NSCLC has been reported. *BRCA1* is a tumor suppressor gene that participates in DNA repair processes; mutations therein elevate the risk of developing breast, ovarian, and other cancers (35). Interestingly, *EGFR*-mutant NSCLC patients with a concurrent germline *BRCA* mutation showed a comparable PFS, and longer OS, in the context of EGFR-TKI treatment compared to patients with the wild-type germline *BRCA* (36).

We also explored the factors associated with EGFR-TKI efficacy in our *EGFR*-mutant SCC cohort. We identified the *CREBBP* mutation, *ZNF217* mutation, and *GRM8* mutation. *CREBBP* is a tumor suppressor gene that encodes a histone modifier, and the *CREBBP* mutation is considered a driver mutation in SCLC (37, 38). Pilot study indicated that *CREBBP* mutation may be involved in mutation profile that fitted Big Bang cancer evolution model (39). *ZNF217* is an oncogene that has deleterious effects in various human cancers (40). Overexpression of the *ZNF217* protein is associated with the development of spontaneous lung or node metastases in mice (41). In NSCLC, the positive expression rate of *ZNF217* protein was higher in cancer tissues than that in paracancerous tissues, and increased with the increase of TMN stage. Poorer OS and PFS were noted in NSCLCs with positive *ZNF217 (* 42). *GRM8* is a member of the G-protein coupled receptors for the glutamate family. Mutations in *GRM8* are reported in 8–16% of lung SCC patients (43, 44). *GRM8* activation promotes lung SCC survival by inhibiting the cAMP pathway and activating the MAPK pathway (45); this agrees with our observations that patients with mutant *GRM8* tended to show greater responsiveness to EGFR-TKI treatment. In the pathway analysis, poorer PFS was observed in the Wnt-mutant subgroup of the *EGFR*-mutant SCC cohort. The *CTNNB1* gene, a key driver of Wnt signaling pathway activity, encodes the β-catenin protein, which regulates cellular proliferation (46). Mutations in *CTNNB1* may lead to tumor proliferation and thus a poorer outcome. In our study, the presence of a Wnt pathway mutation was correlated with reduced responsiveness to EGFR-TKI treatment (47).

TMB, although with debates, has been employed to predict the beneficial population of ICIs. Recent meta-analysis compared the efficacy among first-line ICIs versus standard chemotherapy in TMB high and low patients. After analyzing eight different cohorts from five randomized controlled phase III studies (3848 patients), they found a proven benefit in OS in favor of IO agents in the TMB-high population (48). We did not identify significantly higher TMB in our *EGFR*-mutant SCC patients compared to those with adenocarcinoma, consistent with previous reports (24, 49). Some previous studies (50, 51) found that TMB in SCC was higher than that in adenocarcinoma, however, they did not distinguish between patients with *EGFR* mutations and those without *EGFR* mutations. Herein, the TMB of *EGFR*-mutant SCC seemed to be higher than that of adenocarcinoma, but no significant difference showed. This implied that as long as the patients harbored *EGFR* driver mutations, regardless of lung SCC or adenocarcinoma, the outcome for the mutation number was similar. On the contrary, the TMB was significantly higher in *EGFR* wild-type SCC than *EGFR*-mutant SCC in our study. Actually, the incidence of *EGFR* driver mutations in SCC is very low, which has little effect on the whole TMB value of all SCC patients. The higher TMB in *EGFR*-mutant SCC indicated that *EGFR* driver mutations had a great impact on mutation number of tumor cells, although they belonged to a same disease subtype in pathology. This phenomenon implied that the tumor evolutionary trajectories may had some difference between *EGFR* wild-type SCC and *EGFR*-mutant SCC. Of course, on the other hand, our sample size was limited. More studies with larger sample size are needed to further verify these findings. In addition, tissue specimen-based TMB analysis is frequently constrained by the inadequate tissue volume and tissue quality. Pilot study have confirmed the successful use of cytological samples for TMB analysis (52) and large-scale prospective studies are warranted.

With regard to our study, some limitations need to be acknowledged. First, our *EGFR*-mutated SCC cohort was relatively small, although multicenter recruitment has been undertaken to increase the cohort size. The prevalence of *EGFR* mutation is rather limited in SCC. Second, all of the patients in our study had advanced SCC or adenocarcinoma, diagnosed based on analysis of small samples rather than surgical resection. Third, although we have performed comprehensive genomic characterization of *EGFR*-mutant squamous cell lung cancer and try to figure out why *EGFR*-mutant SCC confers poor responsiveness to TKI in terms of mutation profiles, the underlying mechanisms have not been verified *in vitro* and *in vivo*. Between *EGFR*-mutant SCC and *EGFR*-mutant adenocarcinoma, the observation of similar genomic patterns presents, we are unable to adequately identify the unambiguous mechanism underlying the blunted responsiveness. However, we identified several aberrations as predictors for shortened PFS in SCC patients, and these associations are warranted to be validated in other cohort or *via* experimental approaches.

In conclusion, our study showed that *EGFR*-mutant lung SCC patients responded poorly to EGFR-TKI treatment compared to the *EGFR*-mutant adenocarcinoma patients. In lung SCC, *EGFR* mutation concomitant with a *CREBBP*, *ZNF217*, or Wnt pathway mutation was negatively associated with PFS. Conversely, a mutation in *GRM8* was associated with improved PFS. These findings provide shed light on the mechanism underlying the blunted reactivity to EGFR-TKIs in SCC, and identify *EGFR*-mutant SCC patients as a subgroup likely to benefit from treatment with these agents.

## Data Availability Statement

The original contributions presented in the study are included in the article/**Supplementary Material**. Further inquiries can be directed to the corresponding authors.

## Ethics Statement

The studies involving human participants were reviewed and approved by the institutional review board of Second Affiliated Hospital of Zhejiang University School of Medicine. The patients/participants provided their written informed consent to participate in this study.

## Author Contributions

HS, WL, and YX contributed to study conception and design. RJ, LP, JiaS, JW, YJ, FeiL, JZ, QL, BZ, FenL, and LX conducted patient recruitment and data collection. MW, XW, JY, and YS conducted DNA sequencing and bioinformatics analysis. RJ, QL, and YX drafted the manuscript. All authors contributed to the article and approved the submitted version.

## Funding

This work was supported by Zhejiang Provincial Natural Science Foundation [LY20H010004] and the National Natural Science Foundation of China [81870022].

## Conflict of Interest

MW, XW, JY and YS were employed by Geneseeq Technology Inc.

The remaining authors declare that the research was conducted in the absence of any commercial or financial relationships that could be construed as a potential conflict of interest.

## Supplementary Material

Supplementary Figure 1 Associations of gender **(A)**, smoking status **(B)**, *NF1* **(C)**, *ATR* **(D)**, *BRCA1* mutation, tumor mutation **(E)** burden (TMB) **(F)**, and chromosome instability (CIS) **(G)** with EGFR-TKI efficacy in *EGFR*-mutant squamous cell carcinoma (SCC).
