# Supplementary figures and images for: EGFR-Mutated Squamous Cell Lung Cancer and Its Association With Outcomes
Source: Front Oncol. 2021 Jun 14;11:680804. doi: 10.3389/fonc.2021.680804 (PMC8236808; doi:10.3389/fonc.2021.680804)

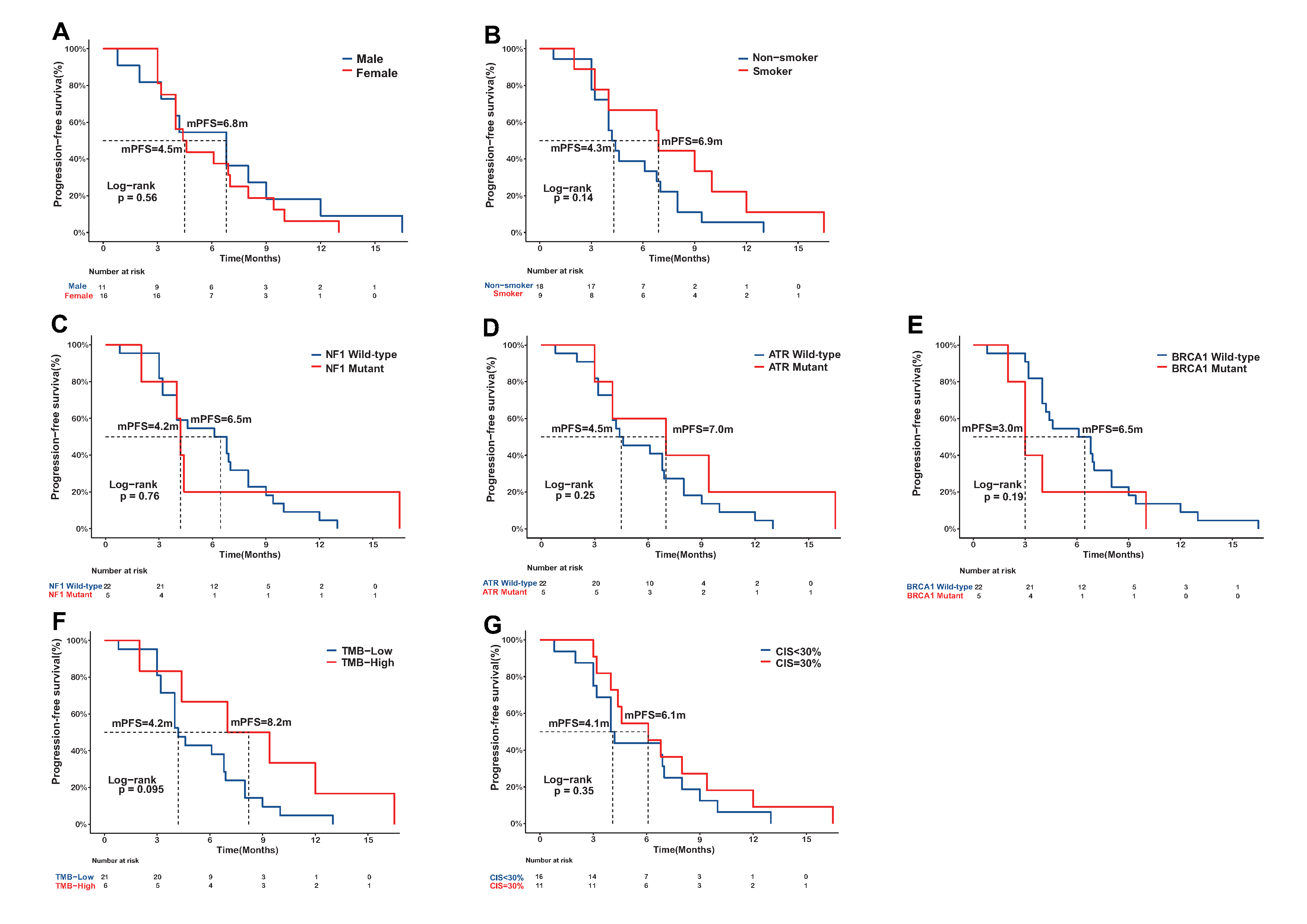

Supplement: Supplementary Figure 1 — Associations of gender (A), smoking status (B), NF1 (C), ATR (D), BRCA1 mutation, tumor mutation (E) burden (TMB) (F), and chromosome instability (CIS) (G) with EGFR-TKI efficacy in EGFR-mutant squamous cell carcinoma (SCC). [file Image_1.tiff]
