# Supplementary material for: EGFR-Mutated Squamous Cell Lung Cancer and Its Association With Outcomes
Source: Front Oncol. 2021 Jun 14;11:680804. doi: 10.3389/fonc.2021.680804 (PMC8236808; doi:10.3389/fonc.2021.680804)
Supplement: Supplementary file 2 [file Table_1.docx]

**Table S1.** 425 cancer-related genes in targeted next-generation sequencing

| *ABCB1 (MDR1)* | *CDC73* | *ERCC2* | *IDH2* | *MTOR* | *PRKACA* | *SOX14* |
| --- | --- | --- | --- | --- | --- | --- |
| *ABCB4* | *CDH1* | *ERCC3* | *IFNG* | *MUTYH* | *PRKACG* | *SOX2* |
| *ABCC2 (MRP2)* | *CDK10* | *ERCC4* | *IFNGR1* | *MYC* | *PRKAR1A* | *SOX21* |
| *ADH1A* | *CDK12* | *ERCC5* | *IGF1R* | *MYCL* | *PRKCI* | *SPOP* |
| *ADH1B* | *CDK4* | *ESR1* | *IGF2* | *MYCN* | *PRKDC* | *SPRY4* |
| *ADH1C* | *CDK6* | *ETV1* | *IKBKE* | *MYD88* | *PRSS1* | *SRC* |
| *AIP* | *CDK8* | *ETV4* | *IKZF1* | *MYH9* | *PRSS3* | *SRY* |
| *AKT1* | *CDKN1A* | *ETV6* | *IL7R* | *NAT1* | *PTCH1* | *STAG2* |
| *AKT2* | *CDKN1B* | *EWSR1* | *INPP4B* | *NBN* | *PTEN* | *STAT3* |
| *AKT3* | *CDKN1C* | *EXT1* | *IRF2* | *NCOR1* | *PTK2* | *STK11* |
| *ALDH2* | *CDKN2A* | *EXT2* | *JAK1* | *NF1* | *PTPN11* | *STMN1* |
| *ALK* | *CDKN2B* | *EZH2* | *JAK2* | *NF2* | *PTPN13* | *STT3A* |
| *AMER1* | *CDKN2C* | *FANCA* | *JAK3* | *NFE2L2* | *PTPRD* | *SUFU* |
| *APC* | *CEBPA* | *FANCC* | *JARID2* | *NFKBIA* | *QKI* | *TAP1* |
| *AR* | *CEP57* | *FANCD2* | *JUN* | *NKX2-1* | *RAC1* | *TAP2* |
| *ARAF* | *CHD4* | *FANCE* | *KDM5A* | *NKX2-4* | *RAC3* | *TEK* |
| *ARID1A* | *CHEK1* | *FANCF* | *KDM6A* | *NOTCH1* | *RAD50* | *TEKT4* |
| *ARID1B* | *CHEK2* | *FANCG* | *KDR (VEGFR2)* | *NOTCH2* | *RAD51* | *TERC* |
| *ARID2* | *CREBBP* | *FANCI* | *KEAP1* | *NOTCH3* | *RAD51B* | *TERT* |
| *ARID5B* | *CRKL* | *FANCL* | *KIF1B* | *NPM1* | *RAD51C* | *TET2* |
| *ASCL4* | *CSF1R* | *FANCM* | *KIF5B* | *NQO1* | *RAD51D* | *TGFBR2* |
| *ASXL1* | *CTCF* | *FAT1* | *KIT* | *NRAS* | *RAD54L* | *THADA* |
| *ATF1* | *CTLA4* | *FBXW7* | *KITLG* | *NRG1* | *RAF1* | *TMEM127* |
| *ATIC* | *CTNNB1* | *FGF19* | *KLLN* | *NSD1* | *RARA* | *TMPRSS2* |
| *ATM* | *CUL3* | *FGFR1* | *KMT2A (MLL)* | *NTRK1* | *RARG* | *TNFAIP3* |
| *ATR* | *CUX1* | *FGFR2* | *KMT2B* | *NTRK2* | *RASGEF1A* | *TNFRSF11A* |
| *ATRX* | *CXCR4* | *FGFR3* | *KMT2C* | *NTRK3* | *RB1* | *TNFRSF14* |
| *AURKA* | *CYLD* | *FGFR4* | *KMT2D (MLL2)* | *PAK3* | *RECQL4* | *TNFRSF19* |
| *AURKB* | *CYP19A1* | *FH* | *KRAS* | *PALB2* | *RELN* | *TNFSF11* |
| *AXIN2* | *CYP2A13* | *FLCN* | *LHCGR* | *PALLD* | *RET* | *TOP1* |
| *AXL* | *CYP2A6* | *FLT1 (VEGFR1)* | *LMO1* | *PARK2* | *RHOA* | *TOP2A* |
| *B2M* | *CYP2A7* | *FLT3* | *LRP1B* | *PARP1* | *RICTOR* | *TP53* |
| *BAD* | *CYP2B6*6* | *FLT4* | *LYN* | *PARP2* | *RNF43* | *TP63* |
| *BAI3* | *CYP2C19*2* | *FOXA1* | *LZTR1* | *PAX5* | *ROS1* | *TPMT* |
| *BAK1* | *CYP2C9*3* | *FOXP1* | *MAP2K1 (MEK1)* | *PBRM1* | *RPTOR* | *TSC1* |
| *BAP1* | *CYP2D6* | *FRG1* | *MAP2K2 (MEK2)* | *PDCD1 (PD1)* | *RRM1* | *TSC2* |
| *BARD1* | *CYP3A4*4* | *GATA1* | *MAP2K4* | *PDCD1LG2 (PD-L2)* | *RUNX1* | *TSHR* |
| *BAX* | *CYP3A5* | *GATA2* | *MAP3K1* | *PDE11A* | *RUNX1T1* | *TTF1* |
| *BCL2* | *DAXX* | *GATA3* | *MAP3K4* | *PDGFRA* | *SBDS* | *TUBB3* |
| *BCL2L11 (BIM)* | *DDR2* | *GATA4* | *MAP4K3* | *PDGFRB* | *SDC4* | *TUBB4A* |
| *BCR* | *DENND1A* | *GATA6* | *MAX* | *PDK1* | *SDHA* | *TUBB4B* |
| *BIRC3* | *DHFR* | *GNA11* | *MCL1* | *PGR* | *SDHB* | *TUBB6* |
| *BLM* | *DICER1* | *GNAQ* | *MDM2* | *PHOX2B* | *SDHC* | *TYMS* |
| *BMPR1A* | *DLL3* | *GNAS* | *MDM4* | *PIK3C3* | *SDHD* | *U2AF1* |
| *BRAF* | *DNMT3A* | *GRIN2A* | *MECOM* | *PIK3CA* | *SEPT9* | *UGT1A1* |
| *BRCA1* | *DPYD* | *GRM3* | *MED12* | *PIK3R1* | *SETBP1* | *VAMP2* |
| *BRCA2* | *DUSP2* | *GRM8* | *MEF2B* | *PIK3R2* | *SETD2* | *VEGFA* |
| *BRD4* | *EGFR* | *GSTM1* | *MEN1* | *PKHD1* | *SF3B1* | *VHL* |
| *BRIP1* | *EML4* | *GSTM4* | *MET* | *PLAG1* | *SGK1* | *WAS* |
| *BTG2* | *EP300* | *GSTM5* | *MGMT* | *PLK1* | *SLC34A2* | *WISP3* |
| *BTK* | *EPAS1* | *GSTP1* | *MITF* | *PMS1* | *SLC3A2* | *WRN* |
| *BUB1B* | *EPCAM* | *GSTT1* | *MLH1* | *PMS2* | *SLC7A8* | *WT1* |
| *c11orf30* | *EPHA2* | *HDAC2* | *MLH3* | *POLD1* | *SMAD2* | *XPA* |
| *CASP8* | *EPHA3* | *HDAC9* | *MLLT1* | *POLD3* | *SMAD3* | *XPC* |
| *CBL* | *EPHA5* | *HGF* | *MLLT3* | *POLE* | *SMAD4* | *XRCC1* |
| *CBLB* | *EPHB2* | *HLA-A* | *MLLT4* | *POLH* | *SMAD7* | *YAP1* |
| *CCND1* | *ERBB2 (HER2)* | *HNF1A* | *MPL* | *POT1* | *SMARCA4* | *ZNF2* |
| *CCNE1* | *ERBB2IP* | *HNF1B* | *MRE11A* | *PPARD* | *SMARCB1* | *ZNF217* |
| *CD274 (PD-L1)* | *ERBB3* | *HRAS* | *MSH2* | *PPP2R1A* | *SMO* | *ZNF703* |
| *CD74* | *ERBB4* | *HSD3B1* | *MSH6* | *PRDM1* | *SOS1* |  |
| *CDA* | *ERCC1* | *IDH1* | *MTHFR* | *PRF1* | *SOX1* |  |
