# Supplementary material for: EGFR-Mutated Squamous Cell Lung Cancer and Its Association With Outcomes
Source: Front Oncol. 2021 Jun 14;11:680804. doi: 10.3389/fonc.2021.680804 (PMC8236808; doi:10.3389/fonc.2021.680804)
Supplement: Supplementary file 4 [file Table_3.docx]

**Table S3.** Comparison of genetic alterations among *EGFR*-mutant adenocarcinoma, *EGFR*-mutant SCC, and *EGFR* wild-type SCC (Fisher exact test).

| Gene | *EGFR*-mutant SCC vs. *EGFR* wild-type SCC | | *EGFR*-mutant SCC vs. *EGFR*-mutant adenocarcinoma | |
| --- | --- | --- | --- | --- |
|  | OR (95%CI) | P value | OR (95%CI) | P value |
| *ATR* | 4.04 (0.60-45.75) | 0.115 | 8.44 (0.87-420.30) | 0.037* |
| *BRCA1* | 4.04 (0.60-45.75) | 0.115 | 8.44 (0.87-420.30) | 0.037* |
| *CHEK1* | 0.84 (0.12-4.80) | 1.000 | Inf (0.62-Inf) | 0.063 |
| *FAT1* | 0 (0-0.54) | 0.004* | 0 (0-7.80) | 0.510 |
| *FBXW7* | 0.68 (0.10-3.58) | 0.727 | Inf (0.62-Inf) | 0.063 |
| *GNAS* | 0 (0-1.50) | 0.073 | 0 (0-7.80) | 0.510 |
| *GRM8* | 1.47 (0.18-11.89) | 0.684 | Inf (0.62-Inf) | 0.063 |
| *KEAP1* | 0 (0-1.50) | 0.073 | 0 (0-57.06) | 1.000 |
| *KMT2A* | 0 (0-3.43) | 0.263 | 0 (0-1.54) | 0.075 |
| *KMT2B* | 0 (0-7.61) | 0.508 | 0 (0-1.17) | 0.074 |
| *NF1* | 4.04 (0.60-45.75) | 0.115 | 8.44 (0.87-420.30) | 0.037* |
| *NFE2L2* | 0.15 (0-1.25) | 0.071 | 1.47 (0.2-119.04) | 1.000 |
| *SMAD4* | 0 (0-1.14) | 0.039* | 0 (0-7.80) | 0.510 |
| *TP53* | 0.20 (0.02-1.23) | 0.057 | 1.88 (0.56-7.02) | 0.291 |
| *ZNF217* | Inf (0.61- (Inf) | 0.065 | Inf (0.62-Inf) | 0.063 |
| *SOX2*_CNV | 0.06 (0.001-0.47) | 0.001* | Inf (0.038-Inf) | 0.406 |
| *EGFR*_CNV | 5.68 (1.23-36.46) | 0.020* | 4.28 (1.03-21.60) | 0.028* |
| *PIK3CA*_CNV | 0 (0-0.36) | <0.001* | NA | NA |
| *ZNF703*_CNV | 0 (0-1.50) | 0.073 | NA | NA |

EGFR: Epidermal growth factor receptor

SCC: Squamous cell carcinoma

OR: Odds ratio

CI: Confidence interval

NA: Not applicable

Inf: Infinity
