# Supplementary material for: EGFR-Mutated Squamous Cell Lung Cancer and Its Association With Outcomes
Source: Front Oncol. 2021 Jun 14;11:680804. doi: 10.3389/fonc.2021.680804 (PMC8236808; doi:10.3389/fonc.2021.680804)
Supplement: Supplementary file 5 [file Table_4.docx]

**Table S4**. Comparison of PFS among *EGFR*-mutant adenocarcinoma and SCC according to altered genes that showed different incidence between *EGFR*-mutant adenocarcinoma and SCC.

| Gene | SCC | | | | | Adenocarcinoma | | |
| --- | --- | --- | --- | --- | --- | --- | --- | --- |
|  | PFS | | HR (95%CI) | P value | PFS | | HR (95%CI) | P value |
| *ATR* WT | 4.5 | 0.54 (0.18~1.59) | | 0.257 | 11.0 | | NE | NE |
| *ATR* Mutant | 7.0 |  | |  | 7.0 | |  |  |
| *BRCA1* WT | 6.5 | 1.97 (0.72~5.37) | | 0.177 | 19.0 | | NE | NE |
| *BRCA1* Mutant | 3.0 |  | |  | 11.0 | |  |  |
| *NF1* WT | 6.5 | 0.84 (0.28~2.49) | | 0.750 | 11.0 | | NE | NE |
| *NF1* Mutant | 4.2 |  | |  | 7.0 | |  |  |
| *EGFR CNV* WT | 5.9 | 0.77 (0.33~1.79) | | 0.538 | 11.6 | | 2.46 (0.97~6.25) | 0.051 |
| *EGFR CNV* | 6.5 |  | |  | 6.9 | |  |  |

WT: Wild-type

SCC: Squamous cell carcinoma

PFS: Progression-free survival

HR: Hazard ratio

CI: Confidence interval

CNV: Copy number variation

NE: Not evaluation
