# Supplementary material for: EGFR-Mutated Squamous Cell Lung Cancer and Its Association With Outcomes
Source: Front Oncol. 2021 Jun 14;11:680804. doi: 10.3389/fonc.2021.680804 (PMC8236808; doi:10.3389/fonc.2021.680804)
Supplement: Supplementary file 6 [file Table_5.docx]

**Table S5.** Comparison of signaling pathways among *EGFR*-mutant adenocarcinoma, EGFR-mutant SCC, and EGFR wild-type SCC (Fisher exact test).

| Gene | *EGFR*-mutant SCC vs. *EGFR* wild-type SCC | | *EGFR*-mutant SCC vs. *EGFR*-mutant adenocarcinoma | |
| --- | --- | --- | --- | --- |
|  | OR (95%CI) | P value | OR (95%CI) | P value |
| RTK-RAS | Inf (1.85-Inf) | 0.004* | NA | NS |
| HIPPO | 0.10 (0.002-0.77) | 0.011* | 0.47 (0.01-6.27) | 0.641 |
| NRF2 | 0.16 (0.02-0.83) | 0.017* | 0.97 (0.08-9.14) | 1.000 |
| TGF-Beta | 0.28 (0.05-1.23) | 0.078 | 0.70 (0.10-3.68) | 0.729 |
| NOTCH | 0.46(0.15-1.36) | 0.143 | 1.07 (0.34-3.27) | 1.000 |
| TP53 | 0.32 (0.03-2.44) | 0.220 | 1.23 (0.28-6.40) | 1.000 |
| Cell.Cycle | 0.59 (0.20-1.73) | 0.330 | 1.01 (0.33-3.01) | 1.000 |
| PI3K | 0.59 (0.18-1.86) | 0.430 | 1.61 (0.55-4.84) | 0.461 |
| MYC | 0.48 (0.08-2.29) | 0.505 | 0.87(0.12-4.93) | 1.000 |
| WNT | 1.54(0.36-6.57) | 0.532 | 0.53 (0.14-1.78) | 0.291 |

EGFR: Epidermal growth factor receptor

SCC: Lung squamous cell carcinoma

OR: Odds ratio

CI: Confidence interval

NS: No Significance

NA: Not applicable

Inf: Infinity
