# Supplementary material for: EGFR-Mutated Squamous Cell Lung Cancer and Its Association With Outcomes
Source: Front Oncol. 2021 Jun 14;11:680804. doi: 10.3389/fonc.2021.680804 (PMC8236808; doi:10.3389/fonc.2021.680804)
Supplement: Supplementary file 7 [file Presentation_1.pdf]

Material and Methods

DNA extraction, library preparation, and targeted enrichment

Genomic DNA from the white blood cells were extracted using the DNeasy Blood & Tissue Kit (Qiagen) and used as the normal control to remove germline variations. FFPE samples were de-paraffinized with xylene, and genomic DNA was extracted using the QIAamp DNA FFPE Tissue Kit (Qiagen). DNA was quantified by Qubit 3.0 using the dsDNA HS Assay Kit (Life Technologies), and the quality was evaluated by a Nanodrop 2000 (Thermo Fisher).

Libraries were prepared by KAPA Hyper Prep kit (KAPA Biosystems), as previously described (1). Briefly, above 50 ng of genomic DNA was sheared into ~350 bp fragments using a Covaris M220 instrument. End repair, A-tailing, and adaptor ligation of fragmented DNA were performed using the KAPA Hyper DNA Library Prep kit (Roche Diagnostics), followed by size selection with Agencourt AMPure XP beads (Beckman Coulter). DNA Libraries were then amplified by polymerase chain reaction (PCR) and purified using Agencourt AMPure XP beads.

Customized xGen lockdown probes panel (Integrated DNA Technologies) were used to selectively enrich for 425 predefined cancer-related genes (Geneseeq Prime panel) (see Supplemental Table S1). Human cot-1 DNA (Life Technologies) and xGen Universal Blocking Oligos (Integrated DNA Technologies) were added as blocking reagents. The capture reaction was performed with Dynabeads M-270 (Life Technologies) and the xGen Lockdown Hybridization and Wash kit (Integrated DNA Technologies). Captured libraries were subjected to PCR amplification with KAPA HiFi HotStart ReadyMix (KAPA Biosystems). The purified library was quantified using the KAPA Library Quantification kit (KAPA Biosystems), and its fragment size distribution was analyzed using a Bioanalyzer 2100.

Sequencing and Bioinformatics Analysis

Target enriched libraries were sequenced on the HiSeq4000 platform (Illumina) with 2×150 bp pair-end reads. Sequencing data were demultiplexed by bcl2fastq (v2.19), analyzed by Trimmomatic (2) to remove low-quality (quality<15) or N bases.

Then the data were aligned to the hg19 reference human genome with the Burrows-Wheeler Aligner (bwa-mem) (3) and further processed using the Picard suite (available at: https://broadinstitute.github.io/picard/) and the Genome Analysis Toolkit (GATK) (4). SNPs and indels were called by VarScan2 (5) and HaplotypeCaller/ UnifiedGenotyper in GATK, with the mutant allele frequency (MAF) cutoff as 0.5%. Common variants were removed using dbSNP and the 1000 Genome project. Germline mutations were filtered out by comparing to patient's whole blood controls.

Gene fusions were identified by FACTERA (6) and copy number variations (CNVs) were analyzed with ADTEx (7). The log2 ratio cut-off for copy number gain was defined as 2.0 for tissue samples. A log2 ratio cut-off of 0.6 was used for copy number loss detection. Allele-specific CNVs were analyzed by FACETS (8) with a 0.2 drift cut-off for unstable joint segments. Chromosome instability score (CIS) was defined as the proportion of the genome with aberrant (purity-adjusted segment-level copy number >=3 or <=1) segmented copy number. TMB was defined as the number of somatic, coding, base substitution, and indel mutations per megabase of genome examined, and was calculated as previously described (9). Briefly, all base substitutions, including non-synonymous and synonymous alterations, and indels in the coding region of targeted genes were considered with the exception of known hotspot mutations in oncogenic driver genes and truncations in tumor suppressors. Synonymous mutations were counted in order to reduce sampling noise and known driver mutations were excluded as they are over-represented in the Panel.

Reference:

1. Yang Z, Yang N, Ou QX, Xiang Y, Jiang T, Wu X, et al. Investigating Novel Resistance Mechanisms to Third-Generation EGFR Tyrosine Kinase Inhibitor Osimertinib in Non-Small Cell Lung Cancer Patients. *Clin Cancer Res* (2018) 24(13):3097-107. doi: 10.1158/1078-0432.Ccr-17-2310. PubMed PMID: WOS:000437270800016.
2. Bolger AM, Lohse M, Usadel B. Trimmomatic: a flexible trimmer for Illumina

sequence data. *Bioinformatics* (2014) 30(15):2114-20. doi: 10.1093/bioinformatics/btu170. PubMed PMID: WOS:000340049100004.

3. Li H, Durbin R. Fast and accurate short read alignment with Burrows-Wheeler transform. *Bioinformatics* (2009) 25(14):1754-60. doi: 10.1093/bioinformatics/btp324. PubMed PMID: WOS:000267665900006.

4. DePristo MA, Banks E, Poplin R, Garimella KV, Maguire JR, Hartl C, et al. A framework for variation discovery and genotyping using next-generation DNA sequencing data. *Nat Genet* (2011) 43(5):491-+. doi: 10.1038/ng.806. PubMed PMID: WOS:000289972600023.

5. Koboldt DC, Zhang QY, Larson DE, Shen D, McLellan MD, Lin L, et al. VarScan 2: Somatic mutation and copy number alteration discovery in cancer by exome sequencing. *Genome Res* (2012) 22(3):568-76. doi: 10.1101/gr.129684.111. PubMed PMID: WOS:000300962600016.

6. Newman AM, Bratman SV, Stehr H, Lee LJ, Liu CL, Diehn M, et al. FACTERA: a practical method for the discovery of genomic rearrangements at breakpoint resolution. *Bioinformatics* (2014) 30(23):3390-3. doi: 10.1093/bioinformatics/btu549. PubMed PMID: WOS:000345827400014.

7. Amarasinghe KC, Li J, Halgamuge SK. CoNVEX: copy number variation estimation in exome sequencing data using HMM. *Bmc Bioinformatics* (2013) 14. doi: Artn S2
10.1186/1471-2105-14-S2-S2. PubMed PMID: WOS:000314468200002.

8. Shen RL, Seshan VE. FACETS: allele-specific copy number and clonal heterogeneity analysis tool for high-throughput DNA sequencing. *Nucleic Acids Res* (2016) 44(16). doi: ARTN e131
10.1093/nar/gkw520. PubMed PMID: WOS:000384687000001.

9. Fang WF, Ma YX, Yin JNC, Hong SD, Zhou HQ, Wang A, et al. Comprehensive Genomic Profiling Identifies Novel Genetic Predictors of Response to Anti-PD-(L)1 Therapies in Non-Small Cell Lung Cancer. *Clin Cancer Res* (2019) 25(16):5015-26. doi: 10.1158/1078-0432.Ccr-19-0585. PubMed PMID: WOS:000481912400019.
